# PriMath—The Role of Intrinsic Factors in Quantitative Cognitive Performance of Highly Social Primates

**DOI:** 10.1002/ece3.73382

**Published:** 2026-04-13

**Authors:** Kata Anna Bán, Ádám Lőrincz, Kata Frei, Fruzsina Cseh, Fanni Pécsy, István Elek Maák

**Affiliations:** ^1^ Department of Ecology University of Szeged Szeged Hungary; ^2^ Doctoral School of Biology University of Szeged Szeged Hungary; ^3^ Doctoral School of Environmental Sciences University of Szeged Szeged Hungary; ^4^ Szeged Zoo Szeged Hungary; ^5^ Museum and Institute of Zoology, Polish Academy of Sciences Warsaw Poland

**Keywords:** age‐related differences, *Callithrix geoffroyi*, quantity discrimination, ratio‐dependency, *Saguinus bicolor*, sex differences

## Abstract

Mechanisms by which animals acquire, process, store, and use information from their environment (i.e., cognitive abilities), like other traits, evolve in response to selection pressures. Individuals with distinct intrinsic traits may benefit to varying degrees from different strategies when solving ecologically relevant cognitive tasks. This effect may be especially pronounced in social species, where group composition can shape the development of different cognitive strategies. In this study, we investigated the quantity discrimination abilities of two highly social New World primates using spontaneous choice tests. Our aim was to explore the effects of species‐specific biology and intrinsic traits, such as sex and age, on cognitive performance. We tested 14 focal individuals using five quantity combinations with varying ratios (0.25, 0.5, 0.75): 1 vs. 2, 1 vs. 4, 3 vs. 4, 6 vs. 8, and 6 vs. 12. We found that performance was primarily driven by ratio‐dependence rather than absolute differences between quantities. This, combined with the lack of significant differences in response time across quantity combinations, suggests that the Approximate Number System (ANS) is the primary cognitive mechanism underlying quantity discrimination in the studied species. Additionally, response time varied with intrinsic traits such as species, sex, and age; however, we did not detect differences in performance. Our results may reflect the unique social structure of these species, as well as differences in their biology, such as feeding behavior, providing valuable insights into the quantitative abilities of New World monkeys. These findings highlight the need for a deeper exploration of the evolutionary trade‐offs and selective pressures shaping the complex interactions among behavior, intrinsic traits, and cognitive performance.

## Graphical abstract

Our study investigated the quantity discrimination ability in a highly social group of New World monkeys, with the goal of understanding how individual differences in performance due to group composition might be explained from an evolutionary perspective. Our results revealed that discrimination was mainly ratio‐dependent, supporting the role of Approximate Number System (ANS) as the primary cognitive mechanism underlying this ability in the studied species. Differences in decision‐making response time were influenced by species‐level factors and individual intrinsic traits, such as sex or age.

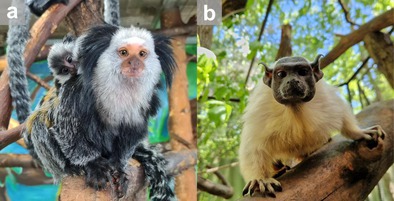

## 1 Introduction

Since researchers began to explore the “animal mind”, numerical cognition (i.e., the ability to represent, discriminate, and process numerical information; Nieder 2020) has held a central place in comparative cognition research, alongside spatial abilities, communication, and tool‐use and manufacture (Vauclair 1996; Beran et al. 2015). The fundamental question is whether animals can perceive and represent the numerical properties of stimuli in the same way they process other characteristics, such as shape, texture, color, or size. Because numbers are more abstract than most physical attributes, it was long assumed that only humans can operate with them (Barnard et al. 2013; Rugani et al. 2014; Beran et al. 2015). This assumption was disproved by several experimental studies, demonstrating that many animals, like humans, are also capable of numerical processing (Cantlon and Brannon 2007; Rugani et al. 2014). This ability is ecologically relevant in various contexts including foraging, navigation, hunting, or social interactions (for an overview, see Nieder 2020), where animals use numerical information to adjust their behavior accordingly (VanMarle et al. 2006). Such numerical abilities have been documented across a wide range of species, both in the wild and under laboratory conditions, including invertebrates (Franks et al. 2015; Bortot et al. 2021), fish (Agrillo et al. 2008; Lucon‐Xiccato et al. 2017), amphibians (Uller et al. 2003; Lucon‐Xiccato et al. 2018), reptiles (Gazzola et al. 2018; Szabo et al. 2021), birds (Farnsworth and Smolinski 2006; Rugani et al. 2014), carnivores (MacNulty et al. 2014; Benson‐Amram et al. 2018), and primates (Boysen and Hallberg 2000; Piantadosi and Cantlon 2017). However, the functional significance of numerical cognitive abilities may vary, particularly in social species, where group size and cohesion can shape how individuals use and rely on numerical information.

To investigate the properties and mechanisms of numerical cognition, generally two methods are applied: spontaneous choice tests and training‐based procedures (Agrillo and Bisazza 2014). In this study, we focus on spontaneous choice tests, in which subjects are presented with two sets of biologically relevant stimuli (usually food or social companions) that differ in numerosity. If individuals can discriminate among quantities in a spontaneous way, they are expected to select the option that provides the greatest benefit, for example, choosing the larger quantity of food to maximize intake (Beran et al. 2015; Agrillo et al. 2017). A key challenge in such experiments is that the numerical properties of biological stimuli naturally co‐vary with continuous quantitative features, such as cumulative size, density, volume, or mass. Traditionally, it was considered that animals use numerosity as a last resort; hence, during experiments, it may be necessary to consider and control for these components (Davis and Pérusse 1988). However, this assumption has been challenged by numerous studies that controlled for continuous quantities and still found that animals could make decisions based on numerical information alone, demonstrating that many species can represent and respond to numerosity naturally, even when co‐varying factors are present (Jordan and Brannon 2006; Cantlon and Brannon 2007; Beran et al. 2008; Gross et al. 2009; Beran 2012).

Two core systems have been identified as responsible for nonverbal numerical representations (for reviews, see Feigenson et al. 2004; Hauser and Spelke 2004). The Approximate Number System (ANS) allows for the estimation of approximate numerosity in large sets of objects or events. It functions without an upper limit, but its accuracy decreases in proportion to increasing magnitude (Hauser and Spelke 2004). The ANS follows Weber's law, which states that the ability to discriminate between two quantities is ratio‐dependent, with smaller ratios (i.e., more distinct differences) enabling easier discrimination (Jordan and Brannon 2006). ANS has been identified as the primary system involved in quantity judgments in a wide range of trained animals (from primates to insects), as well as in spontaneous choice tasks in several vertebrate species (Nieder 2020). Moreover, evidence suggests that the ANS operates similarly in both trained and untrained animals, and even in humans (Hauser and Spelke 2004; Cantlon and Brannon 2006). By contrast, the Object‐Tracking System (OTS)—the second cognitive system—is more controversial, particularly in relation to its function in non‐human animals. This system allows for the exact representation of small quantities in an unconscious and automatic manner (Addessi et al. 2008). Although highly precise, its capacity is limited to small sets (typically up to four items; Barnard et al. 2013; Rugani and Regolin 2022).

Cognitive abilities (i.e., mechanisms that enable animals to acquire, process, store, and use information from their environment, including perception, memory, learning, and decision‐making; Shettleworth 2010), like other traits, evolve in response to selection pressures (Sherry 2006). Selection can favor individuals who perform better in ecologically relevant cognitive tasks, particularly when the challenges they face differ (Lucon‐Xiccato 2022). One prominent line of research investigates sex differences in cognition, where divergent reproductive roles often drive differences in cognitive performance and problem‐solving (Lucon‐Xiccato and Bisazza 2016a, 2016b). Many studies examining sex differences have focused on spatial abilities (e.g., learning new pathways or spatial memory), as differences in spatial behavior frequently result in unequal fitness benefits between males and females (Jones et al. 2003; Lucon‐Xiccato 2022). However, emerging evidence suggests that sex differences may extend beyond spatial tasks, appearing in other cognitive domains, including numerical cognition (Lucon‐Xiccato et al. 2017, 2024; Keagy et al. 2019; Lucon‐Xiccato 2022).

In this study, we aimed to investigate the quantity discrimination ability of two highly social New World monkeys, with the broader goal of understanding how variation in performance may be shaped by evolutionary and intrinsic factors. Specifically, we sought to examine whether species, sex, and age group influence quantitative discrimination efficiency. To do so, we employed spontaneous choice tests, which are well suited to reveal the natural behavioral repertoire of animals (see Agrillo and Bisazza 2014; Bogale et al. 2014). The spontaneous quantity discrimination of captive Geoffroy's marmosets ( *Callithrix geoffroyi* ) and pied tamarins ( *Saguinus bicolor* ) was examined under semi‐natural conditions that allowed for voluntary participation within their social environment. Subjects were presented with five quantity pairs, using grape pieces as stimuli. The experimental design enabled us to examine two effects described by Weber's law: the distance effect (i.e., discrimination improves as the numerical difference between two quantities increases) and the size effect (i.e., discrimination becomes more difficult as the overall magnitude increases). We hypothesized that both species would follow Weber's law to certain extent, and we expected individuals to successfully discriminate quantity pairs with smaller ratios (e.g., 0.25, 0.5). In contrast, we predicted chance‐level performance for more challenging ratios (e.g., 0.75).

To assess the influence of species and intrinsic traits, we analyzed performance across species, sexes (males vs. females), and also age classes (juveniles vs. adults). Although both species belong to the same family (Callitrichidae; Sussman and Kinzey 1984), we predicted species differences in discrimination accuracy, with marmosets outperforming tamarins. Previous delayed reward studies have shown contrasts in performance between the two genera, which have been attributed to differences in feeding ecology. Primarily gummivorous marmosets, whose diet is more specialized, tended to exhibit greater patience, whereas primarily insectivorous tamarins show higher responsiveness, which may be more beneficial under natural circumstances (Stevens, Hallinan and Hauser et al. 2005; Stevens, Rosati, et al. 2005). Based on this, we predicted that the more patient marmosets would perform better in more complex combinations, whereas the more reactive tamarins might perform less accurately, even in relatively simple combinations. We also expected females to outperform males, as previous studies suggest that females in this primate group are more efficient at acquiring food and solving food‐related problems (Tardif and Richter 1981; Petto and Devin 1988; Box 1997; Yamamoto et al. 2004). Finally, although evidence on age‐related numerical cognition is less available in the literature (at least in the group of primates) and sometimes contradictory (Anderson et al. 2005, 2007), we expected juveniles to perform less accurately than adults due to higher impulsivity and reduced attentiveness (Rosati et al. 2023).

## 2 Methods

### 2.1 Species and Subjects

**FIGURE 1:**
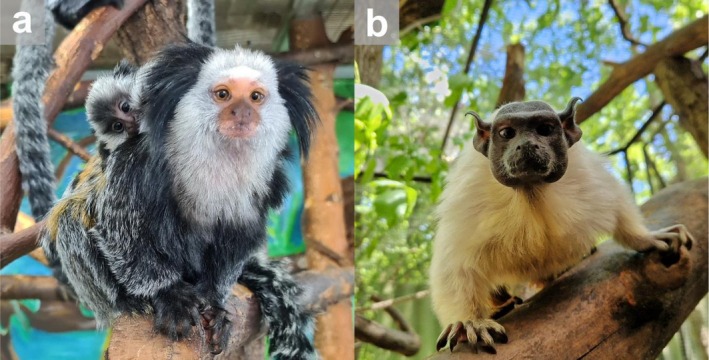
Studied species: (a) Geoffroy's marmoset, *Callithrix geoffroyi* (a father carrying its infant), and (b) pied tamarin, *Saguinus bicolor* .

The studied species, the Geoffroy's marmoset ( *Callithrix geoffroyi* ; Figure 1a) and the pied tamarin ( *Saguinus bicolor* ; Figure 1b), are small, arboreal, group‐living monkeys belonging to the New World primate family Callitrichidae (Platyrrhini). Their main food sources are insects, fruits, and plant exudates; however, representatives of the different genera consume these types of food to different extents, with plant exudates as a primary food source for marmosets and insects for tamarins (Sussman and Kinzey 1984). Social organization and mating system can differ greatly within callitrichines, as monogamy, polygamy, polyandry, and polygynandry have been observed under natural circumstances among them, even within the same genus or population (Fernandez‐Duque et al. 2012). Cooperative breeding is also a characteristic of this group, as fathers and other group members (siblings and even non‐related individuals) contribute to the care of the offspring (Digby et al. 2006; Figure 1a).

The experiment took place in Szeged Zoo, a Hungarian zoological institution (member of the European Association of Zoos and Aquaria—EAZA), where the participants of the study are housed. Altogether, 10 captive‐borne Geoffroy's marmosets ( *C. geoffroyi* ) and four pied tamarins ( *S. bicolor* ) were involved in the study. Male and female representatives of both species were tested, along with four young marmosets (7 and 10 months‐old twin pairs). Monkeys were group‐housed with conspecifics in social units of two or more individuals, consisting of either family groups (a dominant pair with adult, juvenile, and infant offspring of both sexes) or non‐family groups (pairs or single‐sex adult groups). During the testing period, there were no changes in the daily routine of the monkeys. Two meals per day were provided at 8 a.m. and 12 p.m., and they had access to water *ad libitum*. Neither marmosets nor tamarins were involved in any training procedure or experimental study previously.

### 2.2 Apparatus and Experimental Procedure

**FIGURE 2:**
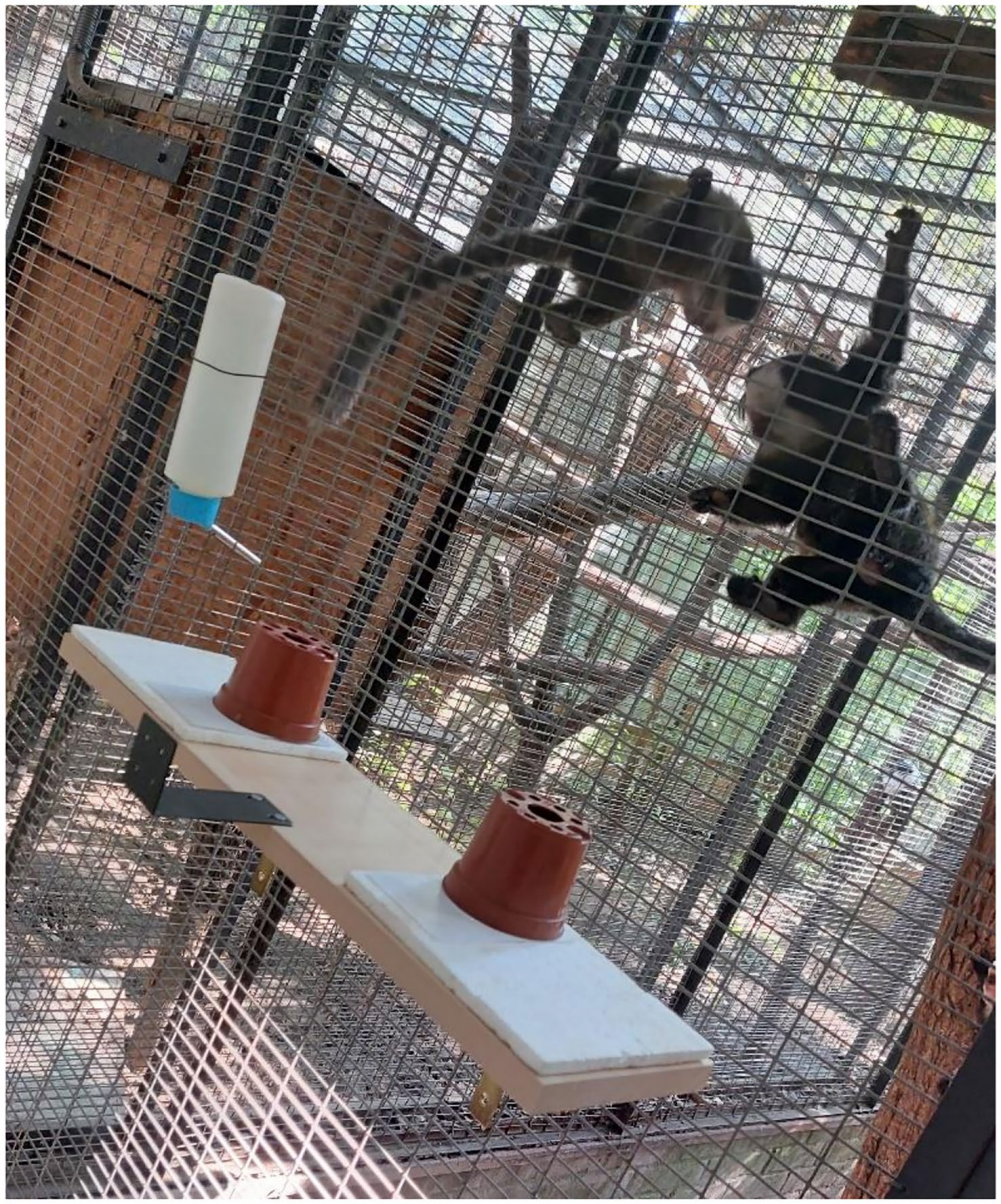
Setup of the experiment with the apparatus and two potential subjects.

The experiment was carried out in 2023 and 2024. In both years, the testing period started in summer (middle of June 2023 and middle of July 2024) and lasted until autumn (middle of September 2023 and beginning of November 2024). Every testing session was held in the same period of the day, between 10 a.m. and 12 p.m. Testing sessions were carried out from the service corridor of the monkeys' outer enclosure where it was possible to get in contact with the subjects through the cage (Figure 2).

To investigate the properties and mechanisms of quantity discrimination, we employed spontaneous choice tests, which are known to reveal the natural behavioral repertoire of species (see Agrillo and Bisazza 2014; Bogale et al. 2014). Equal sized grapes of the same type (1/8 of a grape) were used as stimuli and placed on a movable wooden platform (60 cm × 15 cm × 2 cm) attached to the fence (Figure 2). Grapes were hidden inside opaque containers (brown flowerpots, *d* = 8.5 cm) until the beginning of each trial to prevent pre‐test visual cues from influencing the subjects' choices (Figure 2). Due to the enclosure's layout, individuals could not be separated during testing sessions. Participation was therefore entirely voluntary, resulting in a random and constant exchange of subjects throughout the tests. No individual was forced to participate, and none was tested more than three times consecutively. To avoid losing interest, each session lasted a maximum of 2 h and involved multiple groups and individuals. The number of trials per session varied depending on the monkeys' natural activity rhythms. Prior to the first testing session, subjects underwent a familiarization period with the apparatus to become accustomed to the experimental setup. During this period, the apparatus (wooden platform together with the opaque containers) was placed on the enclosure cage and left there for 2 h a day to allow the animals to become familiar with the apparatus and its elements. We also performed tests with combinations not involved in the testing sessions (e.g., 1 vs. 1) to familiarize them with the procedure.

In our study, we tested five quantity combinations: 1 vs. 2 (0.5), 1 vs. 4 (0.25), 3 vs. 4 (0.75), 6 vs. 8 (0.75), and 6 vs. 12 (0.5). These combinations allowed us to assess discrimination performance across both the small (1–4) and large (> 4) numerical ranges, as well as across small and large numerical ratios. Each combination was presented at least 10 times per individual (min. 50 discrimination trials per subject). Prior to each trial, the designated number of grape pieces was placed separately on both sides of the platform and covered with opaque containers, ensuring that the items did not touch or occlude each other if observed from an overhead perspective. Combinations were presented in a random order, with no combination repeated more than two times consecutively. The side containing the larger (or smaller) set was determined pseudo‐randomly, i.e., randomized across test sessions but balanced equally within each individual. The distance between the cage and the sets of grapes (~10 cm), as well as between the two grape sets (~15 cm), was kept constant by marking the fixed positions of the containers (invisible to the subjects).

During this preparation phase, a black cover was used to prevent subjects from visually detecting the setup. Once the black cover was removed, the subject was guided to position itself between the two containers. Subjects were oriented to the apparatus using a small piece of grape as an incentive, which was withdrawn once they had taken the desired position. When the experimenter (KAB) could confirm that the subject was attentive (i.e., stationary and looking at the experimenter), both containers were lifted simultaneously at the same pace, revealing the two quantities and allowing the subject to choose from the two available options (Video S1). Choice was defined as the behavior when the subject reached through the cage bars and took an item, or, if the item was out of its reach, it either attempted to reach again or refrained from immediately moving toward the alternative option. Subjects were rewarded with one piece of grape for selecting the smaller quantity and with two for selecting the larger. We decided to use this method, as without reinforcement, subjects would have lost motivation to participate. On the other hand, with equal reinforcement, we assumed it would create a bias regarding which option they should choose, as the outcome was always the same.

Each trial was video recorded for subsequent analysis. We considered a trial valid only if the subject inspected both sets and made a clear choice. Trials were excluded if the subject examined only one option (i.e., responding immediately to the first detected set), if other individuals interfered, or if the choice was dubious (e.g., reached between the two sets). Onsite observations were cross‐checked against video recordings for both excluded and valid trials. Ambiguous trials were further evaluated by two independent observers based on the video recordings and were included only upon full agreement regarding the outcome. Because decisions in this experiment were preference‐based and spontaneous, no choice was inherently “correct” or “incorrect”. However, for clarity, we refer to choices in favor of the larger quantity as “correct” and those in favor of the smaller quantity as “incorrect” throughout the text.

Additionally, the time required for decision‐making (response time, in seconds) was quantified from the video recordings as the interval between the removal of the opaque containers and the moment the subject reached through the bars (i.e., made a choice according to our definition) to select one of the options.

### 2.3 Data Analysis

Altogether we used data from 14 individuals (Table 1). We classified all individuals into different groups based on their species (two levels: marmoset and tamarin), sex (two levels: female and male), and age (two levels: juvenile—i.e., falling before the onset of the maturation period, generally 12 months, and adult—i.e., falling after the onset of the maturation period; Yamamoto 1993). For three subjects, we could not complete the 10 repetitions for each combination (Table 2), although they had at least four repetitions for every combination.

**TABLE 1:** Subjects that participated in the experiment and their grouping based on species, sex, and age group. Juvenile individuals are marked with light gray color. Abbreviations: *Cg*– *Callithrix geoffroyi* ; *Sb*— *Saguinus bicolor* ; F—female; M—male; A—adult; J—juvenile.

Subject	Species	Sex	Age group
Lizzy	*Cg*	F	A
Emily	*Cg*	F	A
KisCofi	*Cg*	F	A
Samy	*Cg*	M	A
Winston	*Cg*	M	A
Miguel	*Cg*	M	A
Annie	*Cg*	F	J
Tula	*Cg*	F	J
Stinky	*Cg*	M	J
Danny	*Cg*	M	J
Stella	*Sb*	F	A
Pierre	*Sb*	M	A
Athos	*Sb*	M	A
Porthos	*Sb*	M	A

**TABLE 2:** Number of correct (+) and incorrect (−) choices, as well as the proportion of correct choices, are shown for the five quantity combinations. Significant deviations from the chance level (*p* < 0.05) are indicated in bold, while marginally significant results are in italics. Individuals who did not complete 10 repetitions per combination are highlighted in light gray.

	Combinations										
	1 vs. 2		1 vs. 4		3 vs. 4		6 vs. 8		6 vs. 12		
**Subjects**	+	−	+	−	+	−	+	−	+	−	∑
Lizzy	8	2	9	1	4	6	5	5	5	5	50
Emily	4	7	10	6	5	5	5	5	5	5	57
KisCofi	3	2	10	0	4	3	4	2	5	2	35
Samy	7	3	9	1	6	5	5	5	4	6	51
Winston	6	4	8	2	4	7	6	4	7	3	51
Miguel	6	4	8	2	7	3	6	4	7	3	50
Annie	9	1	8	2	8	3	6	4	6	4	51
Tula	6	4	4	6	5	5	4	6	5	5	50
Stinky	6	4	9	1	4	6	6	4	6	4	50
Danny	5	6	11	0	6	4	8	2	6	4	52
Stella	9	3	10	1	9	3	4	7	5	6	57
Pierre	6	4	7	3	7	3	7	3	9	1	50
Athos	2	2	3	2	2	5	3	5	2	3	29
Porthos	3	4	2	2	4	4	3	5	4	3	34
∑	80	50	108	29	75	62	72	61	76	54	667
**Proportion of correct choices (%)**	61.54		78.83		54.75		54.14		58.46		
z	2.607		4.712		1.109		0.953		1.92		
*p*	**0.009**		**< 0.001**		0.267		0.341		*0.055*		

We used intercept‐only generalized linear mixed‐effects models (GLMMs, binomial error distribution, maximum likelihood fit) to determine whether the proportion of correct choices differed from random chance (50%) in each combination with subject ID included as a random intercept. To determine the factors influencing correct decisions, we also applied GLMMs (binomial error distribution, maximum likelihood fit) with sex and the five quantity combinations included as explanatory factors, the number of repetitions as a covariate, and the subject ID as a random factor. As juveniles from the tamarin species ( *S. bicolor* ) were not tested, we modified the dataset to control for the confounding of age group and species. Therefore, to assess whether species influenced correct decisions, we fitted the GLMM only to adult individuals (*N* = 10) with sex, species, and their interaction as explanatory factors, the number of repetitions as a covariate, and the subject ID as a random factor. Similarly, to assess whether correct decisions were affected by the age group, we fitted the same model structure using only marmoset ( *C. geoffroyi* , *N* = 10) individuals with age group replacing species as an explanatory factor.

To identify the factors influencing the response time of individuals, we used linear mixed‐effects models (LMMs, Gaussian error, maximum likelihood fit) with the same model constructions as described previously for the GLMMs. Non‐significant interaction terms were removed, and the results of the simplified model were reported. Prior to the analyses, response time was log‐transformed to achieve normality. We conducted post‐hoc pairwise comparisons of estimated marginal means (EMMs) across factor levels on both GLMMs and LMMs to see if groups differ significantly.

All statistical analyses were carried out in R version 4.4.2 (R Core Team 2024). LMMs and GLMMs were built using the *lmer* and *glmer* functions of the ‘lme4’ package (Bates et al. 2015). The *Anova* function from the ‘car’ package was used to test model significance (Fox and Weisberg 2019). We used the *AICc* function of the ‘MuMIn’ package to extract the AICc values of the models (Bartoń 2025). Post hoc pairwise comparisons among factor levels were performed with the *emmeans* function (‘emmeans’ package; Lenth 2024).

### 2.4 Ethical Note

As no invasive procedures were conducted, formal ethical approval was not required beyond authorization from the institutional authority of Szeged Zoo, which was granted by the Chief Executive Officer. Data collection was primarily observational: study animals remained within their social groups throughout the study, and their participation in the tests was entirely voluntary. Experiments were performed in accordance with applicable international, national, and/or institutional guidelines for animal welfare, including the guidelines for the use of animals in research issued by the Association for the Study of Animal Behavior (ASAB) and the Animal Behavior Society (ABS; ASAB Ethical Committee/ABS Animal Care Committee 2023).

## 3 Results

**FIGURE 3:**
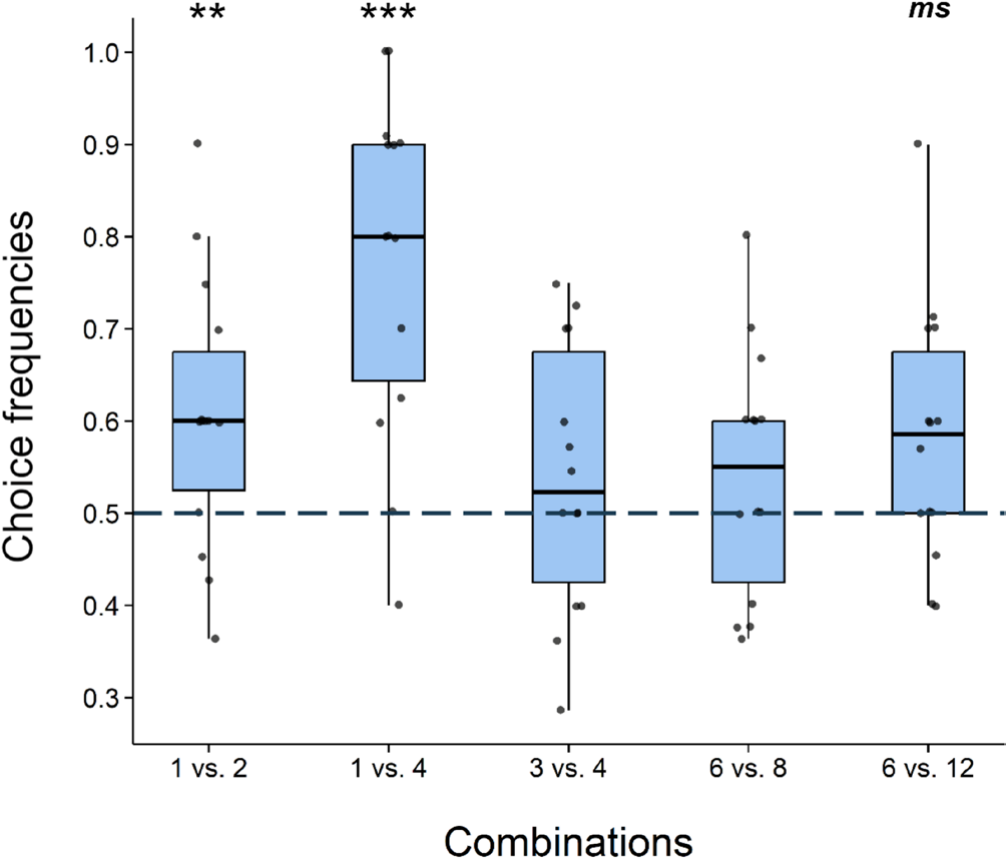
Frequencies of correct choices of individuals (*N* = 14) in relation to the chance level (50%, dashed line). Each point represents the frequency of correct choices of one individual within the combination. Asterisks indicate significant differences between correct and incorrect choices within combinations (*p < 0.05; **p < 0.01; ***p < 0.001). Marginally significant difference (0.05 < *p* < 0.1) between correct and incorrect choices is indicated with *ms*.

Overall, the proportion of correct choices was 61.52% in 1 vs. 2 combination, 78.83% in 1 vs. 4, 54.75% in 3 vs. 4, 54.14% in 6 vs. 8, and 58.46% in 6 vs. 12 (Table 2). Considering all individuals, the probability of choosing the larger food quantity over the smaller in 1 vs. 2 was 61.54% (*N* = 80/130 correct/total choices; 95% CI: 0.529–0.695; *p* = 0.009) and in 1 vs. 4 was 80.85% (*N* = 108/137; 95% CI: 0.699–0.885; *p* < 0.001), indicating a choice probability significantly above chance. In 6 vs. 12, the estimated probability was 58.46% (*N* = 76/130; 95% CI: 0.498–0.666; *p* = 0.055), suggesting a trend toward success above chance, though the interval includes the chance level. This result might be due to the limited sample size; thus, we conducted a simulation‐based power analysis to see the influence of the number of individuals (for more details, see the relevant section in Table S1 and Figure S1). In 3 vs. 4, the probability of choosing the larger amount was 54.74% (*N* = 75/137; 95% CI: 0.464–0.629; *p* = 0.267), and in 6 vs. 8 was 54.14% (*N* = 72/133; 95% CI: 0.456–0.624; *p* = 0.341), implying performance at chance level (Table 2 and Figure 3). Subjects performed significantly better in the 1 vs. 4 combination compared to all other combinations (*z* > 4.223; *p* < 0.001), but performance did not differ in any other comparisons (*z* < 1.194; *p* > 0.755; Table S2).

Correct choices were only influenced by the different quantity combinations (χ^2^ = 22.585; *p* < 0.001), whereas sex (χ^2^ = 0.011; *p* = 0.916) of the individuals and number of repetitions had no significant effect (χ^2^ = 0.0605; *p* = 0.945). Including only adult individuals, we also observed a lack of difference in performance between species (*z* = 0.355; *p* = 0.722), and with only marmosets, we observed the same lack of difference between age groups (*z* = 0.068; *p* = 0.946).

**FIGURE 4:**
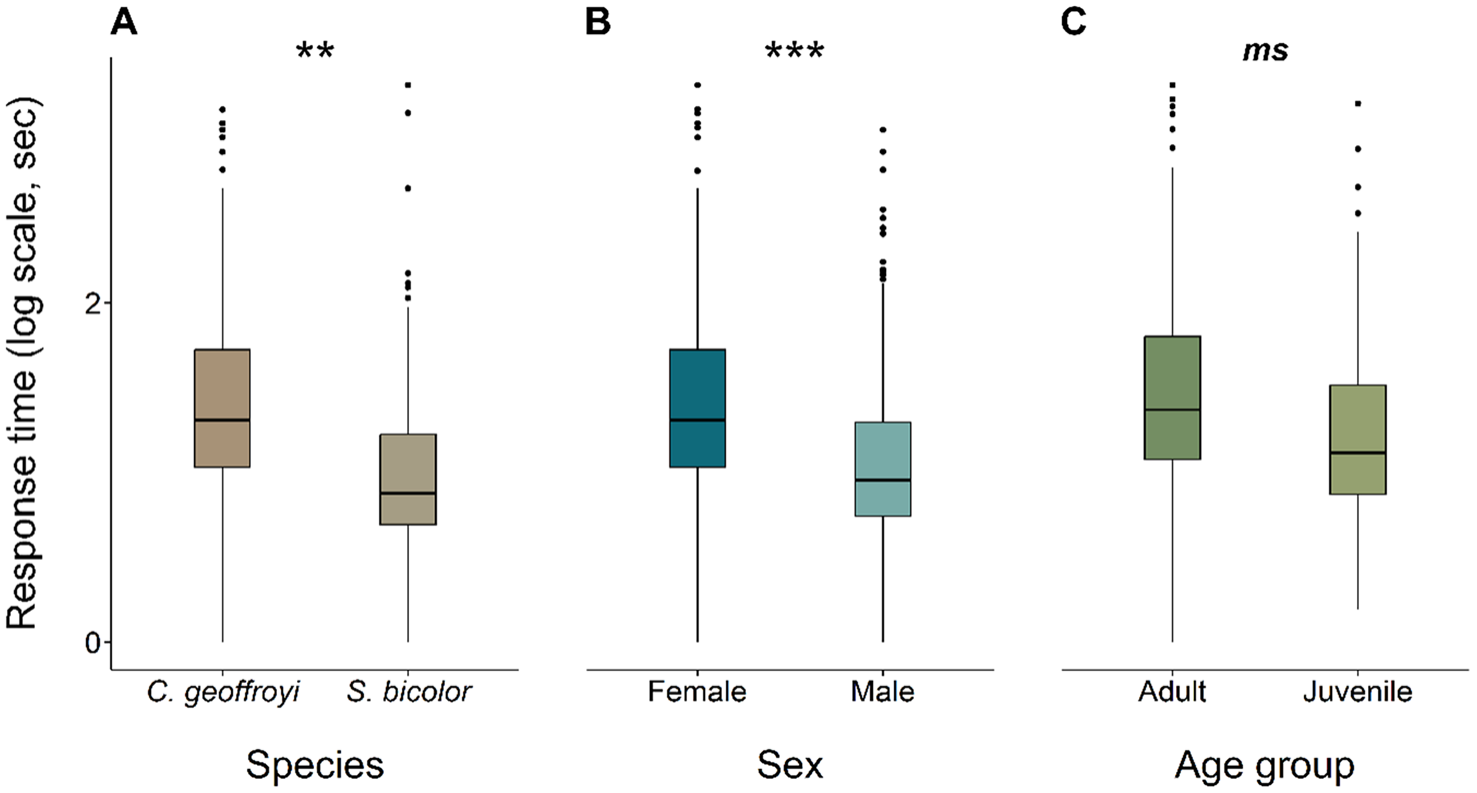
Response time (sec) individuals needed to make a decision according to species (A), sex (B), and age group (C). Asterisks indicate significant differences between different groups (*p < 0.05; **p < 0.01; ***p < 0.001), and a marginally significant difference (0.05 < *p* < 0.1) between groups is indicated with *ms*.

On the other hand, the response time (Tables S3 and S4) was significantly influenced by the sex of the individuals, with faster male response time than female (*t* = 3.284; *p* = 0.007; Figure 4B). Response time decreased with the number of repetitions (*t* = −5.941; *p* < 0.001) but did not differ significantly among the different quantity combinations (*t* < 1.490; *p* > 0.569; Figure S2). With only adult individuals, the interaction between sex and species was not significant (*t* = −1.050; *p* = 0.337), but response time was affected by species with tamarins having faster response time than marmosets (*t* = −3.271; *p* = 0.014; Figure 4A), by sex (*t* = −2.575; *p* = 0.038) with males being faster than females, and was also negatively affected by repetition number (*t* = −4.929; *p* < 0.001). Including only marmosets, similarly, the interaction between sex and the age group had no effect on response time (*t* = 0.978; *p* = 0.367), while males had a faster response time (*t* = −2.710; *p* = 0.031), and repetition number had a negative effect (*t* = −4.223; *p* < 0.001). The effect of age group on response time was marginally significant, with juveniles tending to have faster response time (*t* = −1.943; *p* = 0.093; Figure 4C).

## 4 Discussion

In our study, contrary to our predictions, we could not detect any difference in performance between species, sexes, or age groups, as correct choices were only influenced by the different quantity combinations. Response time, on the other hand, was affected by species and also by intrinsic factors, with significantly longer response times observed in marmosets ( *C. geoffroyi* ), females, and adults. The performance of the investigated monkey species was primarily influenced by the ratio between quantities rather than by their absolute difference. Subjects showed a higher proportion of correct choices with smaller quantities (size effect), while accuracy decreased as the numerical difference between quantities narrowed (distance effect). As the number of repetitions (i.e., progression through the experiment) had no effect on success, although it did affect response time, we can conclude that while individuals acclimated to the task, they did so without evidence of a learning pattern (as incorrect choices still occurred in the final repetitions). Given the ratio‐dependent nature of discrimination success and the absence of significant differences in response time across combinations, our results support that ANS is the primary cognitive mechanism underlying this ability in the studied species.

Although reward magnitude was capped (i.e., subjects got either one or two pieces of grape as a reward and not the full chosen amount), the relative structure of the choice remained the same: subjects chose between visually distinct quantities, and performance followed a ratio‐dependent (Weber‐law) pattern widely interpreted as evidence for ANS functioning. If the observed behavior were driven purely by arbitrary stimulus–reward associations, such systematic ratio effects across pairings would not be expected. The persistence of this pattern despite capped rewards suggests that discrimination relied on approximate magnitude processing rather than solely on reinforcement learning. While we recognize that reward contingencies may influence quantitative performance, our results suggest that this short‐term manipulation did not fundamentally alter the operation of an evolutionarily conserved magnitude‐processing system.

In line with our predictions, individuals discriminated more accurately between quantities with smaller ratios (0.25 and 0.5), namely, 1 vs. 2 and 1 vs. 4, whereas discrimination was more challenging at a larger ratio (0.75), such as 3 vs. 4 and 6 vs. 8. In the 6 vs. 12 combination, we detected only a trend toward above chance performance; however, we believe that a larger sample size would likely provide stronger evidence for a preference for the larger quantity. Thus, further studies including more individuals are needed to clarify this observation. The findings are consistent with the distance effect, which states that when the overall numerical magnitude increases, a larger absolute difference is required for successful discrimination. For example, although 1 vs. 2 and 6 vs. 12 have the same ratio, the proportion of correct choices was higher in the 1 vs. 2 condition. Our results also support the size effect, as higher magnitudes (e.g., 3 vs. 4, 6 vs. 8, and 6 vs. 12) resulted in lower discrimination accuracy compared with lower ones (e.g., 1 vs. 2 and 1 vs. 4). If the OTS had been the dominant mechanism, similarly high performance would have been expected in 1 vs. 2 and 1 vs. 4, given that OTS enables quick and precise quantity judgments at lower magnitudes (Hauser and Spelke 2004; Barnard et al. 2013; Nieder 2020). However, in our experiment, this was not the case. Moreover, the response time data also contradicts the OTS hypothesis: individuals did not complete the “easy” combinations (i.e., 1 vs. 2 and 1 vs. 4) more quickly than the others. Similar patterns have been reported in capuchin monkeys ( *Cebus apella* ; Beran and Parrish 2016) and even in non‐primate species such as domestic cats (*Felis silvestris catus*; Bánszegi et al. 2016). Taken together, our results suggest that the ANS is the primary cognitive mechanism underlying numerical abilities in the studied species.

The observed between‐species pattern, according to which pied tamarins responded more quickly than Geoffroy's marmosets, may be explained by differences in their feeding ecology (Sussman and Kinzey 1984). While both consume similar food sources, they exploit them differently. Marmosets are primarily gummivores, feeding on plant exudates such as gum, sup, and resin, whereas tamarins are mainly insectivores (Sussman and Kinzey 1984; Passamani and Rylands 2000). These dietary differences are reflected in their foraging behavior and may help explain the species‐specific result of the observed response times. Previous research associated with patience and impulse control (e.g., temporal discounting tasks) in closely related species, such as the common marmoset ( *Callithrix jacchus* ) and cotton‐top tamarin ( *Saguinus oedipus* ), has demonstrated considerable variations between species and contexts. Marmosets, in contrast to tamarins, tend to exhibit greater patience, often opting for delayed but larger rewards over immediate, smaller ones (Stevens, Hallinan and Hauser et al. 2005; Stevens, Rosati, et al. 2005). The reason can be that tamarins rely on ephemeral food sources such as insects, where quick decision‐making and impulsive actions are advantageous for successful foraging. On the other hand, marmosets' feeding behavior is more specialized, which also entails some anatomical (e.g., dental) modifications (Sussman and Kinzey 1984; Nash 1986). Since acquiring exudates requires patience, as monkeys must wait for exudates to flow after bark removal (Stevens, Rosati, et al. 2005), this ecological pressure may foster more deliberate and sustained attention. Although we expected species to differ in discrimination accuracy as well, we could not detect a significant difference in this respect.

If the benefits of a given cognitive function differ between sexes in ecologically relevant situations, then selection may favor one sex over the other (Lucon‐Xiccato 2022). Although males and females of the studied species are morphologically similar and behave similarly in many respects, growing evidence suggests sex differences in certain behavioral traits—particularly in feeding behavior—that stem from the peculiarities of their social system (Box 1997; Yamamoto et al. 2004). Michels (1998) found that female common marmosets ( *Callithrix jacchus* ) exhibited greater dominance when food was limited, displaying increased aggression toward group members, particularly males. Females also spent more time searching for food and obtained higher intake. Supporting these findings, other studies on common marmoset ( *C. jacchus* ) and cotton‐top tamarin ( *Saguinus oedipus* ) groups have shown that reproductive females hold a privileged position within their social groups and have priority access to limited food sources (Tardif and Richter 1981; Yamamoto et al. 2004). The reproductive biology of callitrichine females is characterized by twin births and the overlapping energetic demands of pregnancy and lactation, resulting in a high overall energy investment (Box 1997; Digby et al. 2006). This heightened energy demand may explain both their assertive behavior and their dominance over food sources, which ensures constant feeding opportunities for females. Consequently, in such ecological contexts where females generally have secured access to food, there may be reduced evolutionary pressure on them to develop highly accurate quantity discrimination abilities, especially at higher magnitudes. Although we could not reveal a significant difference between females and males in our experiment considering the discrimination accuracy, we expect to detect differences with a higher sample size; thus, additional studies with more individuals are needed to fully explore sex differences connected to quantity discrimination abilities.

Cooperative breeding and male contribution to infant care are characteristics of several New World monkey species, including members of the studied subfamily (Díaz‐Muñoz 2016; Erb and Porter 2017). Observations from both wild and captive populations consistently show that males, particularly dominant ones, are more vigilant than females, likely due to their prominent role in infant care (Rose and Fedigan 1995; Savage et al. 1996; Dolotovskaya and Heymann 2020). Males typically carry infants more often than reproductive females (Goldizen 1987; Garber 1997; Zahed et al. 2010), which not only increases their visibility to predators but also hinders the parent's movement during escape (Tardif 1997; Dolotovskaya and Heymann 2020). A rapid reaction, which we also observed in the response time of males, may represent an adaptive trait shaped by the evolutionary demands of caregiving under risk. As the tested interaction of the sex with either the species or the age group was non‐significant, we can state that, in our study, differences in response time between males and females were not confounded by either factor. However, it is important to note that faster responses do not necessarily imply better numerical discrimination. For instance, in guppies ( *Poecilia reticulata* ), males were significantly quicker in a discrimination learning test, but response time was not correlated with accuracy, suggesting that reaction speed and numerical competence can be dissociated (Lucon‐Xiccato and Bisazza 2016a). Such differences might arise from distinct selection pressures acting on the two sexes (Healy et al. 2009). Although we were unable to incorporate dominance directly into our experimental design, social rank and dominance within and between sexes are highly relevant factors in cognitive research (Milewski et al. 2022). Future studies specifically designed to address the role of dominance in numerical cognition are therefore needed.

While numerical cognitive mechanisms are considered innate, these—like other cognitive skills—can be shaped and improved through experience and learning (Shettleworth 2010; Molina‐García and Barrios 2018; Carvajal and Schuppli 2022). Juveniles possess less life experience than adult conspecifics, and they are generally more prone to distraction and impulsive behavior. Our result suggests a pattern in which juveniles tend to respond faster; however, this finding should be interpreted with caution due to the small number of juveniles in our study and the fact that they belonged to only one species. Testing infant and juvenile primates in different cognitive tasks is crucial for comparative science, especially for theories of human cognitive development, as such studies can help uncover evolutionary foundations (Bjorklund and Pellegrini 2002; Ferrigno et al. 2016). However, cognitive abilities can depend strongly on the pace of development, as the emergence time of specific skills may vary across species due to evolutionary influences (Rosati et al. 2014). To compare skills and abilities across species, we should be aware of this developmental pacing, and gathering evidence about when particular cognitive abilities emerge within a species is therefore fundamental. Studying young New World primate individuals, especially their natural behavior, can be challenging due to their close physical proximity with their parents, which makes individual testing difficult under natural or semi‐natural circumstances. Future studies involving a larger number of juvenile individuals could strengthen our observation of faster response times and help confirm or refute the apparent lack of difference in discrimination performance compared to adults.

In conclusion, the use of spontaneous choice tests allowed us to assess the natural quantitative abilities of two New World monkey species living in social groups under varying ecological pressures. It also enabled us to uncover variation in these abilities and relate them to intrinsic traits such as species, sex, or age group. Our findings suggest that cognitive abilities alone do not fully determine how individuals approach and solve quantitative (or potentially other) cognitive tasks. We believe our study provides valuable insights into the quantitative abilities of New World monkeys and could substantially contribute to comparative literature. Nevertheless, further research is needed to gain a more detailed understanding of the individual differences observed in the quantitative cognition of New World monkeys. Such investigations will facilitate a deeper exploration and understanding of the evolutionary trade‐offs and selective pressures shaping the complex interactions among behavior, intrinsic traits, and cognitive performance.

## Author Contributions

**Kata Anna Bán:** conceptualization (lead), formal analysis (lead), investigation (equal), methodology (lead), visualization (lead), writing – original draft (lead), writing – review and editing (lead). **Ádám Lőrincz:** formal analysis (equal), investigation (equal), writing – review and editing (equal). **Kata Frei:** investigation (equal), writing – review and editing (equal). **Fruzsina Cseh:** investigation (equal), resources (lead), writing – review and editing (equal). **Fanni Pécsy:** investigation (equal), writing – review and editing (equal). **István Elek Maák:** conceptualization (lead), formal analysis (equal), methodology (equal), writing – original draft (equal), writing – review and editing (equal).

## Funding

During the manuscript writing period, IEM was funded by the Bolyai János Scholarship, Hungarian Academy of Sciences (BO/00708/22). KF and KAB were supported by the Academic Excellence Scholarship Program of the Ministry for Culture and Innovation from the source of the National Research, Development and Innovation Fund (KF: EKÖP‐447‐SZTE, KAB: EKÖP‐433‐SZTE). The Open Access publication was supported by the University of Szeged Open Access Fund (ID: 8367).

## Conflicts of Interest

The authors declare no conflicts of interest.

## Supporting information


**Table S1:** Estimated power for each combination with different number of individuals involved (14—actual sample size; 16, 20, and 25—increased, simulated sample sizes).
**Figure S1:** Change of the estimated power with increasing sample size (i.e., number of individuals) for each combination.
**Table S2:** Post hoc pairwise comparison among the five quantity combinations regarding discrimination accuracy. Significant differences (*p* < 0.05) between combinations are indicated with bold face.
**Table S3:** Median response time (s) of individuals for correct (+) and incorrect (−) choices across combinations. Individuals who did not complete 10 repetitions per combination are highlighted in light gray.
**Table S4:** Median response time (s) of different groups for correct (+) and incorrect (−) choices among combinations.
**Figure S2:** Response time (sec) on the logarithmic scale among the five quantity combinations. Lower‐case letters indicate significance among combinations.

**Data S1:** ece373382‐sup‐0002‐Supinfo2‐Code.pdf.

**Data S2:** ece373382‐sup‐0003‐Supinfo3‐Code.rmd.

**Data S3:** ece373382‐sup‐0004‐DataS1.xlsx.

**Video S1:** ece373382‐sup‐0005‐VideoS1.mp4.

## Data Availability

The data associated with this study and the R codes are available as Supporting Information.
