# Supplementary material for: PriMath—The Role of Intrinsic Factors in Quantitative Cognitive Performance of Highly Social Primates
Source: Ecol Evol. 2026 Apr 13;16(4):e73382. doi: 10.1002/ece3.73382 (PMC13071529; doi:10.1002/ece3.73382)
Supplement: Supplementary file 1 — Table S1: Estimated power for each combination with different number of individuals involved (14—actual sample size; 16, 20, and 25—increased, simulated sample sizes). Figure S1: Change of the estimated power with increasing sample size (i.e., number of individuals) for each combination. Table S2: Post hoc pairwise comparison among the five quantity combinations regarding discrimination accuracy. Significant differences (p < 0.05) between combinations are indicated with bold face. Table S3: Median response time (s) of individuals for correct (+) and incorrect (−) choices across combinations. Individuals who did not complete 10 repetitions per combination are highlighted in light gray. Table S4: Median response time (s) of different groups for correct (+) and incorrect (−) choices among combinations. Figure S2: Response time (sec) on the logarithmic scale among the five quantity combinations. Lower‐case letters indicate significance among combinations. [file ECE3-16-e73382-s002.docx]

**Supplementary Material**

**Ecology and Evolution**

PriMath – the role of intrinsic factors in quantitative cognitive performance of highly social primates

Kata Anna Bán^1,2*^, Ádám Lőrincz^1^, Kata Frei^1,3^, Fruzsina Cseh^4^, Fanni Pécsy^1,3^, István Elek Maák^1,5^

^1^Department of Ecology, University of Szeged, Közép fasor 52, 6726 Szeged, Hungary

^2^Doctoral School of Biology, University of Szeged, Közép fasor 52, 6726 Szeged, Hungary

^3^Doctoral School of Environmental Sciences, University of Szeged, Rerrich Béla tér 1, 6720 Szeged, Hungary

^4^Szeged Zoo, Cserepes sor 47, 6725 Szeged, Hungary

^5^Museum and Institute of Zoology, Polish Academy of Sciences, Twarda 51/55, 01-818 Warsaw, Poland

*Corresponding author: [baankataa@gmail.com](mailto:ban.kata.anna@szte.hu)

**Simulation-based power analysis**

Method:

We generated hypothetical datasets using the observed effect size (i.e., the intercept estimates from the intercept-only models on the probability scale) and the original study parameters (i.e., 10 trial/individual). Simulations were conducted for different sample sizes: 14 individuals (corresponding to our actual sample size), and additionally 16, 20, and 25 individuals to assess the effect of increased sample size. For each simulated dataset, we fitted the same mixed-effects model used in the original analysis and estimated the probability of detecting above-chance performance. We performed 1000 simulations per sample size for each combination (5×4000 simulations in total). Statistical power was estimated as the proportion of simulated models resulting a significant effect (i.e., above chance level performance) divided by 1000 (i.e., total number of simulated models).

Results:

**Table S1.** The estimated power for each combination with different number of individuals involved (14 – actual sample size; 16, 20 and 25 – increased, simulated sample sizes).

| **Combination** | **Number of individuals** | **Power estimate** |
| --- | --- | --- |
| 1 vs. 2 | 14 | 0.721 |
|  | 16 | 0.791 |
|  | 20 | 0.895 |
|  | 25 | 0.955 |
| 1 vs. 4 | 14 | 1 |
|  | 16 | 1 |
|  | 20 | 1 |
|  | 25 | 1 |
| 3 vs. 4 | 14 | 0.183 |
|  | 16 | 0.183 |
|  | 20 | 0.270 |
|  | 25 | 0.292 |
| 6 vs. 8 | 14 | 0.155 |
|  | 16 | 0.179 |
|  | 20 | 0.213 |
|  | 25 | 0.239 |
| 6 vs. 12 | 14 | 0.502 |
|  | 16 | 0.540 |
|  | 20 | 0.629 |
|  | 25 | 0.736 |

**
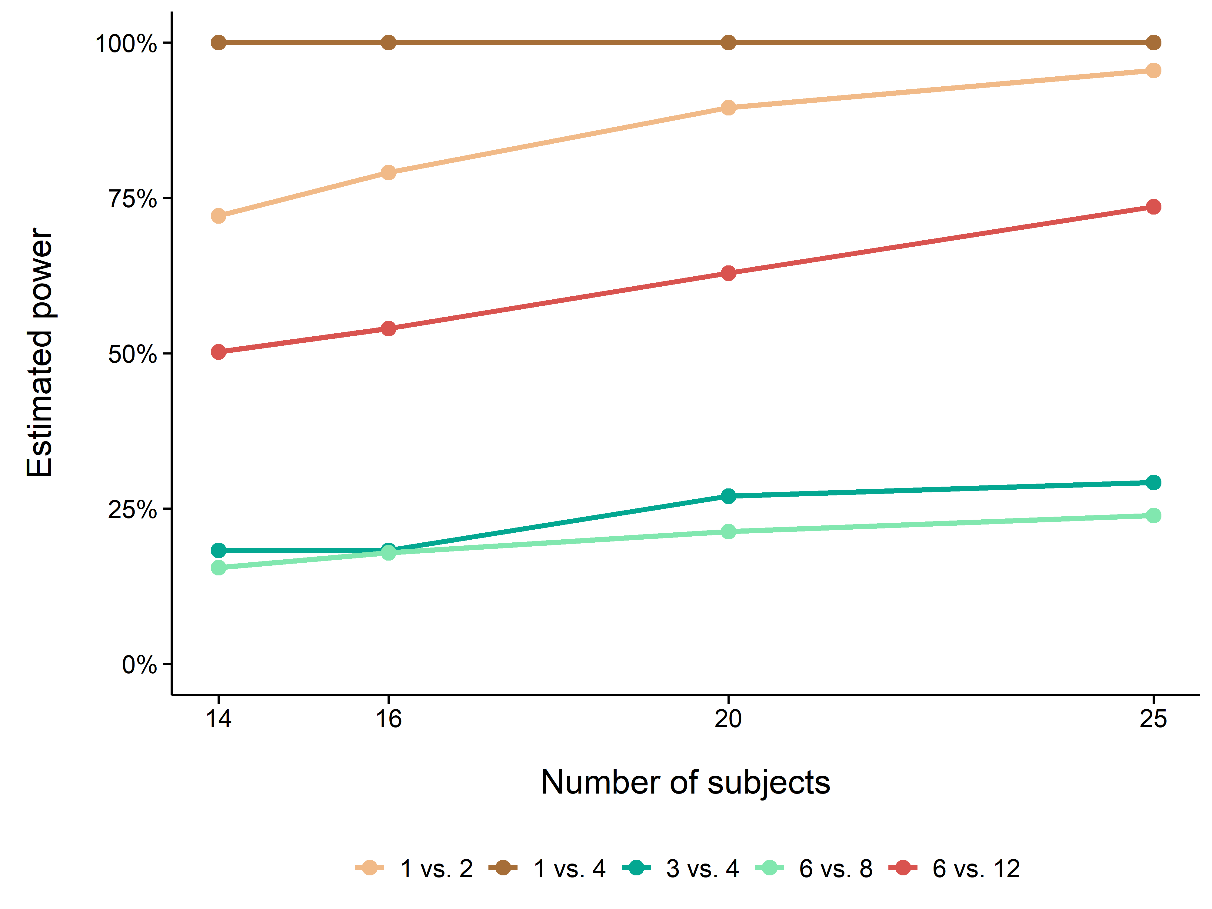
Figure S1.** Change of the estimated power with increasing sample size (i.e., number of individuals) for each combination.

**Table S2.** Post-hoc pairwise comparison among the five quantity combinations regarding discrimination accuracy. Significant differences (*p*<0.05) between combinations are indicated with bold face.

| Comparison | z | *p* |
| --- | --- | --- |
| 1 vs. 2 - 1 vs. 4 | -3.081 | **0.016** |
| 1 vs. 2 - 3 vs. 4 | 1.123 | 0.795 |
| 1 vs. 2 - 6 vs. 8 | 1.194 | 0.755 |
| 1 vs. 2 - 6 vs. 12 | 0.513 | 0.986 |
| 1 vs. 4 - 3 vs. 4 | 4.175 | **<0.001** |
| 1 vs. 4 - 6 vs. 8 | 4.223 | **<0.001** |
| 1 vs. 4 - 6 vs. 12 | 3.568 | **0.003** |
| 3 vs. 4 - 6 vs. 8 | 0.080 | 1.000 |
| 3 vs. 4 - 6 vs. 12 | -0.605 | 0.974 |
| 6 vs. 12 - 6 vs. 8 | 0.680 | 0.961 |

**Table S3.** Median response time (s) of individuals for correct (+) and incorrect (–) choices across combinations. Individuals who did not complete 10 repetitions per combination are highlighted in light grey.

|  | **Median response time (s)** | | | | | | | | | |
| --- | --- | --- | --- | --- | --- | --- | --- | --- | --- | --- |
| **Combinations** | **1 vs. 2** | | **1 vs. 4** | | **3 vs. 4** | | **6 vs. 8** | | **6 vs. 12** | |
| **Subjects** | **+** | **−** | **+** | **−** | **+** | **−** | **+** | **−** | **+** | **−** |
| Lizzy | 3.7 | 2.4 | 3.7 | 2.8 | 4.9 | 3.05 | 4.2 | 4.4 | 3.7 | 5.6 |
| Emily | 2.75 | 3.3 | 3.25 | 4.6 | 3.6 | 3.6 | 4.4 | 3.8 | 2.9 | 5.6 |
| KisCofi | 3.2 | 10.6 | 5.45 | 0 | 4.7 | 3.9 | 7.9 | 16.35 | 9.2 | 6.05 |
| Samy | 2.8 | 2 | 4.2 | 4.2 | 3.55 | 3 | 3.9 | 3.3 | 3.25 | 4.15 |
| Winston | 3 | 4 | 3.25 | 6.75 | 3.75 | 3 | 2 | 2.9 | 1.7 | 2.2 |
| Miguel | 3.55 | 3 | 5.4 | 6.4 | 5 | 6.2 | 4.05 | 6.15 | 3.9 | 2.7 |
| Annie | 4.9 | 3.9 | 4.3 | 3.8 | 3.15 | 6 | 5.05 | 4.95 | 6.55 | 4.25 |
| Tula | 3.2 | 3.55 | 4.9 | 3.8 | 3 | 3.2 | 3 | 3.4 | 2.8 | 3.6 |
| Stinky | 2.65 | 2.6 | 1.8 | 1.8 | 2 | 2.3 | 2.35 | 2.45 | 1.95 | 2.7 |
| Danny | 2.2 | 2.7 | 2.7 | 0 | 3.65 | 2.5 | 2.35 | 2.1 | 2.75 | 2.5 |
| Stella | 4.8 | 1.8 | 2.7 | 5.4 | 3.7 | 5.5 | 2.65 | 2.4 | 3.2 | 2.8 |
| Pierre | 2.6 | 2.2 | 2.3 | 2.6 | 1.8 | 2.4 | 2.2 | 3.4 | 2 | 2.2 |
| Athos | 1.95 | 2.25 | 2 | 3.8 | 2.75 | 2.2 | 1.7 | 2.2 | 2.1 | 2.3 |
| Porthos | 3 | 2.5 | 2.6 | 3.2 | 2.45 | 2.4 | 3 | 2.4 | 2.95 | 2.4 |

**Table S4.** Median response time (s) of different groups for correct (+) and incorrect (–) choices among combinations.

|  | **Median response time (s)** | | | | | | | | | |
| --- | --- | --- | --- | --- | --- | --- | --- | --- | --- | --- |
| **Combinations** | **1 vs. 2** | | **1 vs. 4** | | **3 vs. 4** | | **6 vs. 8** | | **6 vs. 12** | |
| **Grouping** | **+** | **−** | **+** | **−** | **+** | **−** | **+** | **−** | **+** | **−** |
| All individuals | 3.2 | 2.8 | 3.3 | 3.8 | 3 | 2.95 | 3.2 | 3.3 | 2.8 | 3.4 |
| Females | 4.3 | 3.1 | 2.6 | 4.2 | 3.2 | 3.4 | 4.5 | 4.2 | 3.2 | 4.7 |
| Males | 2.9 | 2.5 | 3.6 | 3.85 | 2.75 | 2.6 | 2.4 | 2.7 | 2.2 | 2.6 |
| *S. bicolor* adults | 3.3 | 3.25 | 4 | 5.05 | 3.9 | 3.1 | 4.1 | 4.3 | 3.1 | 4.45 |
| *C. geoffroyi* adults | 3.25 | 2.2 | 2.25 | 3.2 | 2.15 | 2.4 | 2.2 | 2.4 | 2.15 | 2.4 |
| *C. geoffroyi* juveniles | 3.2 | 2.9 | 2.8 | 3.8 | 3 | 3 | 2.8 | 2.65 | 2.7 | 3 |


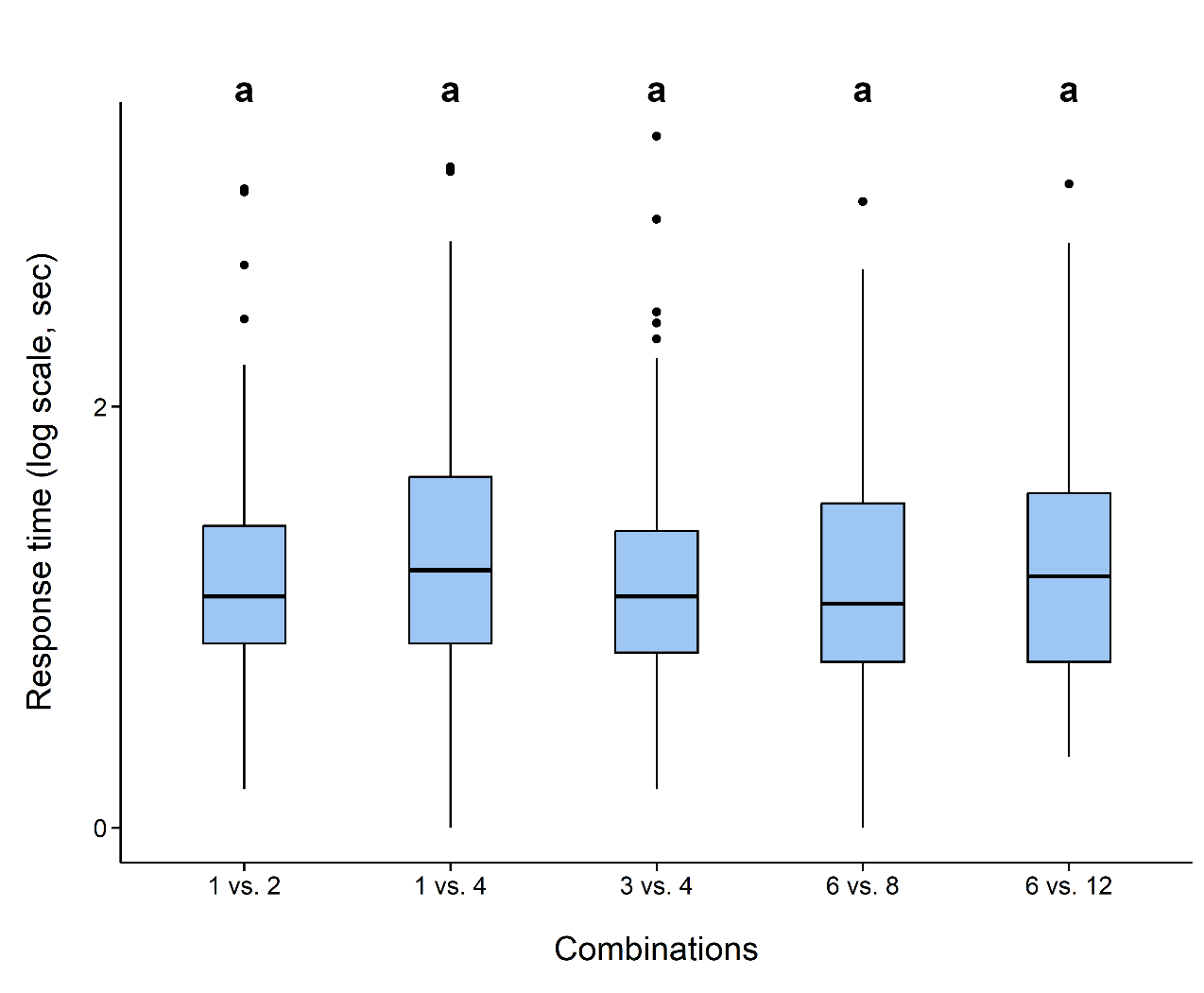


**Figure S2.** Response time (sec) on logarithmic scale among the five quantity combinations. Lower case letters indicate significance among combinations.
