# Supplementary material for: PriMath—The Role of Intrinsic Factors in Quantitative Cognitive Performance of Highly Social Primates
Source: Ecol Evol. 2026 Apr 13;16(4):e73382. doi: 10.1002/ece3.73382 (PMC13071529; doi:10.1002/ece3.73382)
Supplement: Supplementary file 2 — Data S1: ece373382‐sup‐0002‐Supinfo2‐Code.pdf. [file ECE3-16-e73382-s005.pdf]

#package

```r
require(readxl)
require(lme4)
require(car)
require(emmeans)
require(MuMIn)
require(MASS)

data_all=read_excel("D:/num_datasets.xlsx", sheet="all_24_23_clear_REV")
```

#Intercept-only models for each combination

```r
#The fixed intercept is estimated on the logit scale (log-odds). If the
intercept = 0 → probability = 0.5. So testing whether the intercept is
different from 0 is the same as testing whether probability ≠ 0.5.

#1 vs. 2
data_1_2=subset(data_all, Combo=="1_2")
m=glmer(Success ~ 1 + (1|Subject), data = data_1_2, family = binomial)

## boundary (singular) fit: see help('isSingular')

summary(m)

## Generalized linear mixed model fit by maximum likelihood (Laplace
##   Approximation) [glmerMod]
##  Family: binomial  ( logit )
## Formula: Success ~ 1 + (1 | Subject)
##    Data: data_1_2
##
##      AIC       BIC    logLik -2*log(L)  df.resid
##     177.2     183.0     -86.6     173.2       128
##
## Scaled residuals:
##     Min      1Q  Median      3Q     Max
## -1.2649 -1.2649  0.7906  0.7906  0.7906
##
## Random effects:
##  Groups  Name        Variance Std.Dev.
##  Subject (Intercept) 4e-14    2e-07
## Number of obs: 130, groups:  Subject, 14
##
## Fixed effects:
##             Estimate Std. Error z value Pr(>|z|)
## (Intercept)   0.4700     0.1803   2.607  0.00913 **
## ---
## Signif. codes:  0 '***' 0.001 '**' 0.01 '*' 0.05 '.' 0.1 ' ' 1
## optimizer (Nelder_Mead) convergence code: 0 (OK)
## boundary (singular) fit: see help('isSingular')
```

```r
fixef(m) #intercept on logit scale

## (Intercept)
##   0.4700036

confint(m, parm = "(Intercept)", method="Wald") #CI on logit scale

##                 2.5 %    97.5 %
## (Intercept) 0.1166662 0.8233411

plogis(fixef(m)) #intercept on probability scale

## (Intercept)
##   0.6153846

plogis(confint(m, parm = "(Intercept)", method="Wald")) # CI on probability
scale

##                 2.5 %    97.5 %
## (Intercept) 0.5291335 0.6949451

#1 vs. 4
data_1_4=subset(data_all, Combo=="1_4")
m2=glmer(Success ~ 1 + (1|Subject), data = data_1_4, family = binomial)
summary(m2)

## Generalized linear mixed model fit by maximum likelihood (Laplace
##   Approximation) [glmerMod]
##  Family: binomial  ( logit )
## Formula: Success ~ 1 + (1 | Subject)
##    Data: data_1_4
## 
##       AIC       BIC    logLik -2*log(L)  df.resid
##     142.7     148.6     -69.4     138.7       135
## 
## Scaled residuals:
##     Min       1Q   Median       3Q      Max
## -2.4033  0.3564  0.4256  0.4925  0.8110
## 
## Random effects:
##  Groups  Name        Variance Std.Dev.
##  Subject (Intercept) 0.5026   0.7089
## Number of obs: 137, groups:  Subject, 14
## 
## Fixed effects:
##             Estimate Std. Error z value Pr(>|z|)
## (Intercept)   1.4403     0.3056   4.712 2.45e-06 ***
## ---
## Signif. codes:  0 '***' 0.001 '**' 0.01 '*' 0.05 '.' 0.1 ' ' 1

fixef(m2)
```

```
## (Intercept)
##    1.440325
```

```r
confint(m2, parm = "(Intercept)", method="Wald")
```

```
##                 2.5 %   97.5 %
## (Intercept) 0.8412739 2.039375
```

```r
plogis(fixef(m2))
```

```
## (Intercept)
##   0.8085049
```

```r
plogis(confint(m2, parm = "(Intercept)", method="Wald"))
```

```
##                 2.5 %    97.5 %
## (Intercept) 0.6987335 0.8848696
```

```r
#3 vs. 4
data_3_4=subset(data_all, Combo=="3_4")
m3=glmer(Success ~ 1 + (1|Subject), data = data_3_4, family = binomial)
```

```
## boundary (singular) fit: see help('isSingular')
```

```r
summary(m3)
```

```
## Generalized linear mixed model fit by maximum likelihood (Laplace
##   Approximation) [glmerMod]
##  Family: binomial  ( logit )
## Formula: Success ~ 1 + (1 | Subject)
##    Data: data_3_4
##
##       AIC       BIC    logLik -2*log(L)  df.resid
##     192.7     198.5     -94.3     188.7       135
##
## Scaled residuals:
##     Min      1Q  Median      3Q     Max
## -1.0998 -1.0998  0.9092  0.9092  0.9092
##
## Random effects:
##  Groups  Name        Variance Std.Dev.
##  Subject (Intercept) 0        0
## Number of obs: 137, groups:  Subject, 14
##
## Fixed effects:
##             Estimate Std. Error z value Pr(>|z|)
## (Intercept)   0.1904     0.1716   1.109    0.267
## optimizer (Nelder_Mead) convergence code: 0 (OK)
## boundary (singular) fit: see help('isSingular')
```

```r
fixef(m3)
```

```
## (Intercept)
##   0.1903537
```

```r
confint(m3, parm = "(Intercept)", method="Wald")
```

```
##                  2.5 %    97.5 %
## (Intercept) -0.1460662 0.5267737
```

```r
plogis(fixef(m3))
```

```
## (Intercept)
##   0.5474453
```

```r
plogis(confint(m3, parm = "(Intercept)", method="Wald"))
```

```
##                 2.5 %    97.5 %
## (Intercept) 0.4635482 0.6287303
```

```r
#6 vs.8
data_6_8=subset(data_all, Combo=="6_8")
m4=glmer(Success ~ 1 + (1|Subject), data = data_6_8, family = binomial)
```

```
## boundary (singular) fit: see help('isSingular')
```

```r
summary(m4)
```

```
## Generalized linear mixed model fit by maximum likelihood (Laplace
##   Approximation) [glmerMod]
##  Family: binomial  ( logit )
## Formula: Success ~ 1 + (1 | Subject)
##    Data: data_6_8
##
##       AIC       BIC    logLik -2*log(L)  df.resid
##     187.5     193.2     -91.7     183.5       131
##
## Scaled residuals:
##     Min      1Q  Median      3Q     Max
## -1.0864 -1.0864  0.9204  0.9204  0.9204
##
## Random effects:
##  Groups  Name        Variance  Std.Dev.
##  Subject (Intercept) 7.193e-15 8.481e-08
## Number of obs: 133, groups:  Subject, 14
##
## Fixed effects:
##             Estimate Std. Error z value Pr(>|z|)
## (Intercept)   0.1658     0.1740   0.953    0.341
## optimizer (Nelder_Mead) convergence code: 0 (OK)
## boundary (singular) fit: see help('isSingular')
```

```r
fixef(m4)
```

```
## (Intercept)
##   0.1657923

confint(m4, parm = "(Intercept)", method="Wald")

##                  2.5 %    97.5 %
## (Intercept) -0.1752771 0.5068617

plogis(fixef(m4))

## (Intercept)
##   0.5413534

plogis(confint(m4, parm = "(Intercept)", method="Wald"))

##                 2.5 %    97.5 %
## (Intercept) 0.4562926 0.6240705

#6 vs. 12
data_6_12=subset(data_all, Combo=="6_12")
m5=glmer(Success ~ 1 + (1|Subject), data = data_6_12, family = binomial)

## boundary (singular) fit: see help('isSingular')

summary(m5)

## Generalized linear mixed model fit by maximum likelihood (Laplace
##   Approximation) [glmerMod]
##  Family: binomial  ( logit )
## Formula: Success ~ 1 + (1 | Subject)
##    Data: data_6_12
##
##      AIC       BIC    logLik -2*log(L)  df.resid
##     180.5     186.2     -88.2     176.5       128
##
## Scaled residuals:
##     Min      1Q  Median      3Q     Max
## -1.1863 -1.1863  0.8429  0.8429  0.8429
##
## Random effects:
##  Groups  Name        Variance Std.Dev.
##  Subject (Intercept) 0        0
## Number of obs: 130, groups:  Subject, 14
##
## Fixed effects:
##             Estimate Std. Error z value Pr(>|z|)
## (Intercept)   0.3417     0.1780    1.92   0.0548 .
## ---
## Signif. codes:  0 '***' 0.001 '**' 0.01 '*' 0.05 '.' 0.1 ' ' 1
## optimizer (Nelder_Mead) convergence code: 0 (OK)
## boundary (singular) fit: see help('isSingular')
```

```r
confint(m5, parm = "(Intercept)", method="Wald")
```

```
##                    2.5 %    97.5 %
## (Intercept) -0.007082649 0.6905812
```

```r
plogis(fixef(m5))
```

```
## (Intercept)
##   0.5846154
```

```r
plogis(confint(m5, parm = "(Intercept)", method="Wald"))
```

```
##                 2.5 %    97.5 %
## (Intercept) 0.4982293 0.6660962
```

#Effects on success

```r
#original model
m_all=glmer(Success~Combo+Sex+Rep+(1|Subject), data = data_all, family =
binomial)
summary(m_all)
```

```
## Generalized linear mixed model fit by maximum likelihood (Laplace
##   Approximation) [glmerMod]
##  Family: binomial  ( logit )
## Formula: Success ~ Combo + Sex + Rep + (1 | Subject)
##    Data: data_all
##
##       AIC       BIC    logLik -2*log(L)  df.resid
##     877.1     913.1    -430.6     861.1       659
##
## Scaled residuals:
##     Min      1Q  Median      3Q     Max
## -2.1552 -1.1001  0.5162  0.8402  1.0517
##
## Random effects:
##  Groups  Name        Variance Std.Dev.
##  Subject (Intercept) 0.06498  0.2549
## Number of obs: 667, groups:  Subject, 14
##
## Fixed effects:
##              Estimate Std. Error z value Pr(>|z|)
## (Intercept)  0.44472    0.27586   1.612  0.10694
## Combo1_4     0.85803    0.27845   3.081  0.00206 **
## Combo3_4    -0.28171    0.25090  -1.123  0.26152
## Combo6_12   -0.13093    0.25526  -0.513  0.60802
## Combo6_8    -0.30154    0.25258  -1.194  0.23255
## SexM         0.02290    0.21669   0.106  0.91582
## Rep          0.00191    0.02762   0.069  0.94486
## ---
## Signif. codes:  0 '***' 0.001 '**' 0.01 '*' 0.05 '.' 0.1 ' ' 1
##
```

```
## Correlation of Fixed Effects:
##           (Intr) Cmb1_4 Cmb3_4 Cm6_12 Cmb6_8 SexM
## Combo1_4  -0.418
## Combo3_4  -0.468  0.472
## Combo6_12 -0.476  0.463  0.515
## Combo6_8  -0.471  0.469  0.521  0.512
## SexM      -0.468  0.018 -0.006  0.001 -0.011
## Rep       -0.558 -0.037 -0.013  0.012  0.003  0.053
```

```
Anova(m_all)
```

```
## Analysis of Deviance Table (Type II Wald chisquare tests)
##
## Response: Success
##         Chisq Df Pr(>Chisq)
## Combo 22.5329  4   0.000157 ***
## Sex    0.0112  1   0.915824
## Rep    0.0048  1   0.944862
## ---
## Signif. codes:  0 '***' 0.001 '**' 0.01 '*' 0.05 '.' 0.1 ' ' 1
```

```
AICc(m_all)
```

```
## [1] 877.3214
```

```
comparison=pairs(emmeans(m_all,~Combo))
comparison
```

```
##  contrast   estimate    SE  df z.ratio p.value
##  1_2 - 1_4   -0.8580 0.278 Inf  -3.081  0.0176
##  1_2 - 3_4    0.2817 0.251 Inf   1.123  0.7945
##  1_2 - 6_12   0.1309 0.255 Inf   0.513  0.9861
##  1_2 - 6_8    0.3015 0.253 Inf   1.194  0.7550
##  1_4 - 3_4    1.1397 0.273 Inf   4.175  0.0003
##  1_4 - 6_12   0.9890 0.277 Inf   3.568  0.0033
##  1_4 - 6_8    1.1596 0.275 Inf   4.223  0.0002
##  3_4 - 6_12  -0.1508 0.249 Inf  -0.605  0.9744
##  3_4 - 6_8    0.0198 0.246 Inf   0.080  1.0000
##  6_12 - 6_8   0.1706 0.251 Inf   0.680  0.9608
##
## Results are averaged over the levels of: Sex
## Results are given on the log odds ratio (not the response) scale.
## P value adjustment: tukey method for comparing a family of 5 estimates
```

```
#Testing for species difference
data_ad=subset(data_all, Age == "02a")

m_ad=glmer(Success~Sex*Sp+Rep+(1|Subject), family = binomial, na.action =
"na.exclude",data = data_ad)
summary(m_ad)
```

```
## Generalized linear mixed model fit by maximum likelihood (Laplace
##   Approximation) [glmerMod]
##  Family: binomial  ( logit )
## Formula: Success ~ Sex * Sp + Rep + (1 | Subject)
##    Data: data_ad
##
##      AIC       BIC    logLik -2*log(L)  df.resid
##     629.6     654.4    -308.8     617.6       458
##
## Scaled residuals:
##     Min      1Q  Median      3Q     Max
## -1.4618 -1.2093  0.7360  0.7919  0.9971
##
## Random effects:
##  Groups  Name        Variance Std.Dev.
##  Subject (Intercept) 0.04067  0.2017
## Number of obs: 464, groups:  Subject, 10
##
## Fixed effects:
##              Estimate Std. Error z value Pr(>|z|)
## (Intercept)  0.29508    0.27522   1.072    0.284
## SexM         0.08611    0.29435   0.293    0.770
## SpSb         0.14368    0.40422   0.355    0.722
## Rep          0.02927    0.03224   0.908    0.364
## SexM:SpSb   -0.42275    0.50780  -0.833    0.405
##
## Correlation of Fixed Effects:
##           (Intr) SexM   SpSb   Rep
## SexM      -0.548
## SpSb      -0.368  0.375
## Rep       -0.639 -0.006 -0.053
## SexM:SpSb  0.243 -0.573 -0.794  0.107

Anova(m_ad)

## Analysis of Deviance Table (Type II Wald chisquare tests)
##
## Response: Success
##         Chisq Df Pr(>Chisq)
## Sex    0.0509  1     0.8215
## Sp     0.2531  1     0.6149
## Rep    0.8246  1     0.3638
## Sex:Sp 0.6931  1     0.4051

#Testing for age difference
data_Cg=subset(data_all, Sp == "Cg")

m_Cg=glmer(Success~Sex*Age+Rep+(1|Subject), family = binomial, na.action =
"na.exclude",data = data_Cg)
summary(m_Cg)
```

```
## Generalized linear mixed model fit by maximum likelihood (Laplace
##   Approximation) [glmerMod]
##  Family: binomial  ( logit )
## Formula: Success ~ Sex * Age + Rep + (1 | Subject)
##    Data: data_Cg
## 
##       AIC       BIC    logLik -2*log(L)  df.resid 
##     668.8     694.0    -328.4     656.8       491 
## 
## Scaled residuals: 
##     Min      1Q  Median      3Q     Max 
## -1.4609 -1.2411  0.7356  0.7743  0.9121 
## 
## Random effects:
##  Groups  Name        Variance Std.Dev.
##  Subject (Intercept) 0.02025  0.1423  
## Number of obs: 497, groups:  Subject, 10
## 
## Fixed effects:
##             Estimate Std. Error z value Pr(>|z|)  
## (Intercept)  0.51578    0.28530   1.808   0.0706 .
## SexM         0.22875    0.32499   0.704   0.4815  
## Age02a       0.02032    0.29852   0.068   0.9457  
## Rep         -0.01653    0.03090  -0.535   0.5927  
## SexM:Age02a -0.13092    0.42195  -0.310   0.7563  
## ---
## Signif. codes:  0 '***' 0.001 '**' 0.01 '*' 0.05 '.' 0.1 ' ' 1
## 
## Correlation of Fixed Effects:
##             (Intr) SexM   Age02a Rep   
## SexM        -0.555                     
## Age02a      -0.613  0.534              
## Rep         -0.603 -0.007  0.010       
## SexM:Age02a  0.432 -0.770 -0.707 -0.004
```

```
Anova(m_Cg)
```

```
## Analysis of Deviance Table (Type II Wald chisquare tests)
## 
## Response: Success
##          Chisq Df Pr(>Chisq)
## Sex     0.5310  1     0.4662
## Age     0.0459  1     0.8304
## Rep     0.2861  1     0.5927
## Sex:Age 0.0963  1     0.7563
```

#Effects on response time

```
m_time=lmer(Time~Combo+Sex+Rep+(1|Subject), na.action = "na.exclude",data =
data_all)
```

```r
plot(m_time)
```

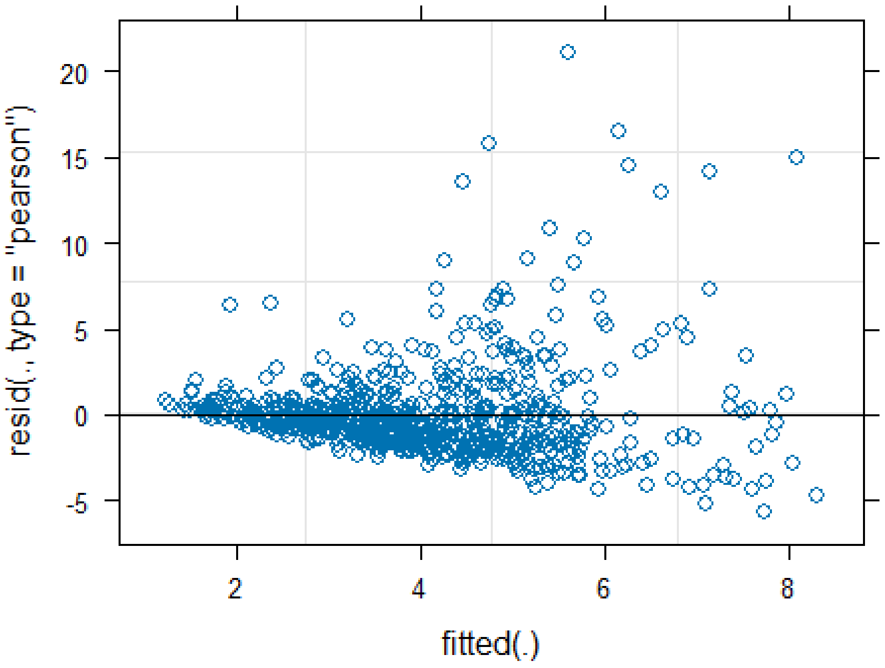


```r
#we log-transformed the data to achieve normality
m_time_log=lmer(log(Time)~Combo+Sex+Rep+(1|Subject), na.action =
"na.exclude",data = data_all)

plot(m_time_log)
```

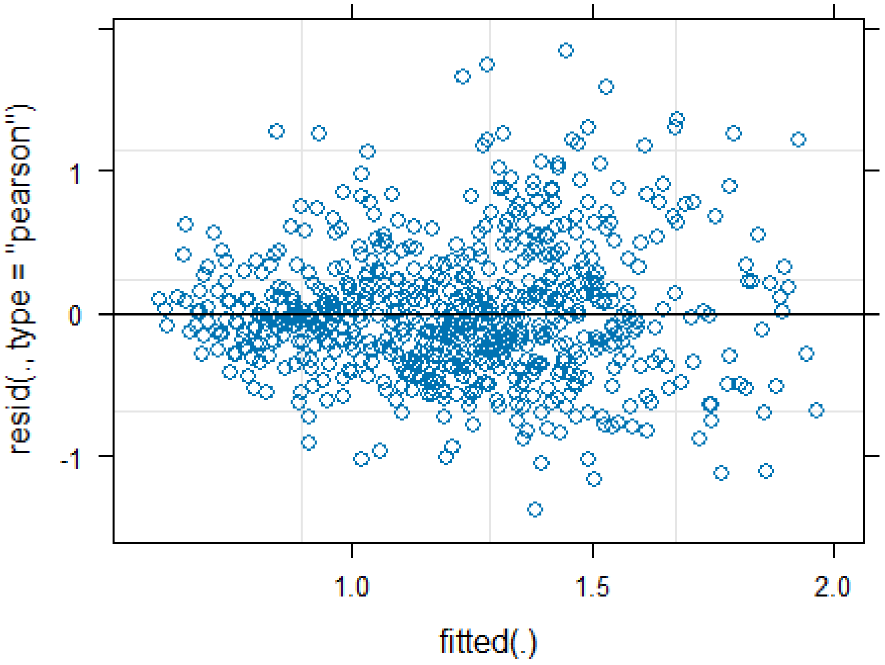


```r
summary(m_time_log)

## Linear mixed model fit by REML ['lmerMod']
## Formula: log(Time) ~ Combo + Sex + Rep + (1 | Subject)
##    Data: data_all
##
## REML criterion at convergence: 961.1
##
## Scaled residuals:
##     Min      1Q  Median      3Q     Max
## -2.8978 -0.6375 -0.1265  0.5027  3.8644
##
## Random effects:
##  Groups   Name        Variance Std.Dev.
##  Subject  (Intercept) 0.04839  0.2200
##  Residual             0.22694  0.4764
## Number of obs: 667, groups:  Subject, 14
##
## Fixed effects:
##              Estimate Std. Error t value
## (Intercept)  1.630491   0.107021  15.235
## Combo1_4     0.071145   0.058505   1.216
## Combo3_4    -0.014885   0.058384  -0.255
## Combo6_12    0.001651   0.059111   0.028
## Combo6_8     0.048263   0.058818   0.821
## SexM        -0.409383   0.124668  -3.284
## Rep         -0.037313   0.006281  -5.941
```

```
##
## Correlation of Fixed Effects:
##            (Intr) Cmb1_4 Cmb3_4 Cm6_12 Cmb6_8 SexM
## Combo1_4  -0.274
## Combo3_4  -0.274  0.512
## Combo6_12 -0.280  0.505  0.507
## Combo6_8  -0.276  0.508  0.510  0.503
## SexM      -0.670  0.009 -0.002  0.001 -0.004
## Rep       -0.322 -0.037 -0.017  0.008 -0.001  0.021
```

```
Anova(m_time_log)
```

```
## Analysis of Deviance Table (Type II Wald chisquare tests)
##
## Response: log(Time)
##          Chisq Df Pr(>Chisq)
## Combo  3.1827  4   0.527723
## Sex   10.7832  1   0.001024 **
## Rep   35.2937  1  2.835e-09 ***
## ---
## Signif. codes:  0 '***' 0.001 '**' 0.01 '*' 0.05 '.' 0.1 ' ' 1
```

```
AICc(m_time_log)
```

```
## [1] 979.4018
```

```
m_time_combo=pairs(emmeans(m_time_log, ~Combo))
```

```
## Registered S3 methods overwritten by 'broom':
##   method         from
##   nobs.fitdistr MuMIn
##   nobs.multinom MuMIn
```

```
m_time_combo
```

```
##  contrast   estimate     SE  df t.ratio p.value
##  1_2 - 1_4  -0.07115 0.0585 649  -1.216  0.7421
##  1_2 - 3_4   0.01489 0.0584 648   0.255  0.9991
##  1_2 - 6_12 -0.00165 0.0591 648  -0.028  1.0000
##  1_2 - 6_8  -0.04826 0.0588 648  -0.821  0.9243
##  1_4 - 3_4   0.08603 0.0577 649   1.490  0.5692
##  1_4 - 6_12  0.06949 0.0585 648   1.188  0.7583
##  1_4 - 6_8   0.02288 0.0582 649   0.393  0.9950
##  3_4 - 6_12 -0.01654 0.0584 648  -0.283  0.9986
##  3_4 - 6_8  -0.06315 0.0580 648  -1.089  0.8125
##  6_12 - 6_8 -0.04661 0.0588 648  -0.793  0.9327
##
## Results are averaged over the levels of: Sex
## Degrees-of-freedom method: kenward-roger
## Results are given on the log (not the response) scale.
## P value adjustment: tukey method for comparing a family of 5 estimates
```

```
m_t_sex=pairs(emmeans(m_time_log,~Sex))
m_t_sex

##  contrast estimate    SE df t.ratio p.value
##  F - M       0.409 0.125 12   3.284  0.0066
##
## Results are averaged over the levels of: Combo
## Degrees-of-freedom method: kenward-roger
## Results are given on the log (not the response) scale.

#Testing for age difference
data_Cg=subset(data_all, Sp == "Cg")

m_time_Cg=lmer(log(Time)~Sex*Age+Rep+(1|Subject), na.action =
"na.exclude",data = data_Cg)
summary(m_time_Cg)

## Linear mixed model fit by REML ['lmerMod']
## Formula: log(Time) ~ Sex * Age + Rep + (1 | Subject)
##    Data: data_Cg
##
## REML criterion at convergence: 733.6
##
## Scaled residuals:
##     Min      1Q  Median      3Q     Max
## -2.3534 -0.6397 -0.1374  0.5271  3.5023
##
## Random effects:
##  Groups   Name        Variance Std.Dev.
##  Subject  (Intercept) 0.02891  0.17
##  Residual             0.24012  0.49
## Number of obs: 497, groups:  Subject, 10
##
## Fixed effects:
##              Estimate Std. Error t value
## (Intercept)  1.579184   0.136001  11.612
## SexM        -0.453941   0.183420  -2.475
## Age02a       0.114117   0.167995   0.679
## Rep         -0.031038   0.007344  -4.227
## SexM:Age02a  0.231989   0.237168   0.978
##
## Correlation of Fixed Effects:
##             (Intr) SexM   Age02a Rep
## SexM        -0.674
## Age02a      -0.739  0.546
## Rep         -0.300 -0.002  0.006
## SexM:Age02a  0.523 -0.773 -0.708 -0.003

Anova(m_time_Cg)
```

```
## Analysis of Deviance Table (Type II Wald chisquare tests)
##
## Response: log(Time)
##             Chisq Df Pr(>Chisq)
## Sex        7.3472  1   0.006717 **
## Age        3.7787  1   0.051908 .
## Rep       17.8638  1  2.373e-05 ***
## Sex:Age    0.9568  1   0.327993
## ---
## Signif. codes:  0 '***' 0.001 '**' 0.01 '*' 0.05 '.' 0.1 ' ' 1
```

```
AICc(m_time_Cg)
```

```
## [1] 747.7954
```

```
m_t_sex=pairs(emmeans(m_time_Cg,~Sex))
```

```
## NOTE: Results may be misleading due to involvement in interactions
```

```
m_t_sex
```

```
##  contrast estimate    SE   df t.ratio p.value
##  F - M       0.338 0.119 5.97   2.850  0.0294
##
## Results are averaged over the levels of: Age
## Degrees-of-freedom method: kenward-roger
## Results are given on the log (not the response) scale.
```

```
m_t_age=pairs(emmeans(m_time_Cg,~Age))
```

```
## NOTE: Results may be misleading due to involvement in interactions
```

```
m_t_age
```

```
##  contrast  estimate    SE   df t.ratio p.value
##  01y - 02a    -0.23 0.119 5.96  -1.940  0.1007
##
## Results are averaged over the levels of: Sex
## Degrees-of-freedom method: kenward-roger
## Results are given on the log (not the response) scale.
```

```
#interaction was non-significant
m_time_Cg2=lmer(log(Time)~Sex+Age+Rep+(1|Subject), na.action =
"na.exclude",data = data_Cg)
summary(m_time_Cg2)
```

```
## Linear mixed model fit by REML ['lmerMod']
## Formula: log(Time) ~ Sex + Age + Rep + (1 | Subject)
##    Data: data_Cg
##
## REML criterion at convergence: 733.5
##
## Scaled residuals:
```

```
##     Min      1Q  Median      3Q     Max 
## -2.3720 -0.6335 -0.1577  0.5193  3.5159 
## 
## Random effects:
##  Groups   Name        Variance Std.Dev.
##  Subject  (Intercept) 0.02893  0.1701  
##  Residual             0.24010  0.4900  
## Number of obs: 497, groups:  Subject, 10
## 
## Fixed effects:
##              Estimate Std. Error t value
## (Intercept)  1.509621   0.115961  13.018
## SexM        -0.315190   0.116321  -2.710
## Age02a       0.230518   0.118625   1.943
## Rep         -0.031013   0.007343  -4.223
## 
## Correlation of Fixed Effects:
##        (Intr) SexM   Age02a
## SexM   -0.499              
## Age02a -0.612 -0.003       
## Rep    -0.350 -0.007  0.006
```

```
Anova(m_time_Cg2)
```

```
## Analysis of Deviance Table (Type II Wald chisquare tests)
## 
## Response: log(Time)
##       Chisq Df Pr(>Chisq)    
## Sex  7.3423  1   0.006735 ** 
## Age  3.7762  1   0.051987 .  
## Rep 17.8367  1  2.407e-05 ***
## ---
## Signif. codes:  0 '***' 0.001 '**' 0.01 '*' 0.05 '.' 0.1 ' ' 1
```

```
AICc(m_time_Cg2)
```

```
## [1] 745.6545
```

```
m_t_sex=pairs(emmeans(m_time_Cg2,~Sex))
m_t_sex
```

```
##  contrast estimate    SE   df t.ratio p.value
##  F - M       0.315 0.116 6.99   2.709  0.0303
## 
## Results are averaged over the levels of: Age 
## Degrees-of-freedom method: kenward-roger 
## Results are given on the log (not the response) scale.
```

```
m_t_age=pairs(emmeans(m_time_Cg2,~Age))
m_t_age
```

```
##  contrast  estimate    SE   df t.ratio p.value
##  01y - 02a   -0.231 0.119 6.97  -1.943  0.0933
##
## Results are averaged over the levels of: Sex
## Degrees-of-freedom method: kenward-roger
## Results are given on the log (not the response) scale.
```

```
anova(m_time_Cg, m_time_Cg2)
```

```
## refitting model(s) with ML (instead of REML)
```

```
## Data: data_Cg
## Models:
## m_time_Cg2: log(Time) ~ Sex + Age + Rep + (1 | Subject)
## m_time_Cg: log(Time) ~ Sex * Age + Rep + (1 | Subject)
##            npar    AIC    BIC  logLik -2*log(L)  Chisq Df Pr(>Chisq)
## m_time_Cg2    6 728.17 753.42 -358.09    716.17
## m_time_Cg     7 728.63 758.09 -357.31    714.63 1.5404  1     0.2146
```

```
#Testing for species difference
data_ad=subset(data_all, Age == "02a")

m_time_ad=lmer(log(Time)~Sex*Sp+Rep+(1|Subject), na.action =
"na.exclude",data = data_ad)
summary(m_time_ad)
```

```
## Linear mixed model fit by REML ['lmerMod']
## Formula: log(Time) ~ Sex * Sp + Rep + (1 | Subject)
##    Data: data_ad
##
## REML criterion at convergence: 694.8
##
## Scaled residuals:
##     Min      1Q  Median      3Q     Max
## -2.6667 -0.6473 -0.1116  0.5605  3.6532
##
## Random effects:
##  Groups   Name        Variance Std.Dev.
##  Subject  (Intercept) 0.02636  0.1624
##  Residual             0.24516  0.4951
## Number of obs: 464, groups:  Subject, 10
##
## Fixed effects:
##              Estimate Std. Error t value
## (Intercept)  1.735088   0.111147  15.611
## SexM        -0.220116   0.144822  -1.520
## SpSb        -0.220367   0.203152  -1.085
## Rep         -0.038870   0.007797  -4.985
## SexM:SpSb   -0.263288   0.250822  -1.050
##
## Correlation of Fixed Effects:
```

```
##            (Intr) SexM   SpSb   Rep
## SexM       -0.654
## SpSb       -0.457  0.360
## Rep        -0.380 -0.009 -0.031
## SexM:SpSb  0.356 -0.578 -0.811  0.061
```

```r
Anova(m_time_ad)
```

```
## Analysis of Deviance Table (Type II Wald chisquare tests)
##
## Response: log(Time)
##           Chisq Df Pr(>Chisq)
## Sex      6.7891  1  0.0091714 **
## Sp      10.9538  1  0.0009341 ***
## Rep     24.8498  1  6.198e-07 ***
## Sex:Sp   1.1019  1  0.2938546
## ---
## Signif. codes:  0 '***' 0.001 '**' 0.01 '*' 0.05 '.' 0.1 ' ' 1
```

```r
AICc(m_time_ad)
```

```
## [1] 709.0654
```

```r
m_t_sp=pairs(emmeans(m_time_ad,~Sp))
```

```
## NOTE: Results may be misleading due to involvement in interactions
```

```r
m_t_sp
```

```
##  contrast estimate    SE   df t.ratio p.value
##  Cg - Sb     0.352 0.125 5.73   2.811  0.0322
##
## Results are averaged over the levels of: Sex
## Degrees-of-freedom method: kenward-roger
## Results are given on the log (not the response) scale.
```

```r
m_t_sex=pairs(emmeans(m_time_ad,~Sex))
```

```
## NOTE: Results may be misleading due to involvement in interactions
```

```r
m_t_sex
```

```
##  contrast estimate    SE   df t.ratio p.value
##  F - M       0.352 0.125 5.76   2.806  0.0323
##
## Results are averaged over the levels of: Sp
## Degrees-of-freedom method: kenward-roger
## Results are given on the log (not the response) scale.
```

```r
#interaction was non-significant
m_time_ad2=lmer(log(Time)~Sex+Sp+Rep+(1|Subject), na.action =
"na.exclude",data = data_ad)
summary(m_time_ad2)
```

```
## Linear mixed model fit by REML ['lmerMod']
## Formula: log(Time) ~ Sex + Sp + Rep + (1 | Subject)
##    Data: data_ad
## 
## REML criterion at convergence: 695
## 
## Scaled residuals: 
##     Min      1Q  Median      3Q     Max 
## -2.6302 -0.6358 -0.1267  0.5707  3.6942 
## 
## Random effects:
##  Groups   Name        Variance Std.Dev.
##  Subject  (Intercept) 0.02712  0.1647  
##  Residual             0.24512  0.4951  
## Number of obs: 464, groups:  Subject, 10
## 
## Fixed effects:
##              Estimate Std. Error t value
## (Intercept)  1.776708   0.104873  16.941
## SexM        -0.308053   0.119622  -2.575
## SpSb        -0.393432   0.120262  -3.271
## Rep         -0.038364   0.007784  -4.929
## 
## Correlation of Fixed Effects:
##      (Intr) SexM   SpSb  
## SexM -0.588              
## SpSb -0.307 -0.229       
## Rep  -0.426  0.032  0.031
```

```
Anova(m_time_ad2)
```

```
## Analysis of Deviance Table (Type II Wald chisquare tests)
## 
## Response: log(Time)
##       Chisq Df Pr(>Chisq)    
## Sex  6.6318  1    0.01002 *  
## Sp  10.7024  1    0.00107 ** 
## Rep 24.2929  1  8.274e-07 ***
## ---
## Signif. codes:  0 '***' 0.001 '**' 0.01 '*' 0.05 '.' 0.1 ' ' 1
```

```
AICc(m_time_ad2)
```

```
## [1] 707.1754
```

```
m_t_sp=pairs(emmeans(m_time_ad2,~Sp))
m_t_sp
```

```
##  contrast estimate   SE   df t.ratio p.value
##  Cg - Sb     0.393 0.12 6.91   3.270  0.0139
## 
```

```
## Results are averaged over the levels of: Sex
## Degrees-of-freedom method: kenward-roger
## Results are given on the log (not the response) scale.
```

```
m_t_sex=pairs(emmeans(m_time_ad2,~Sex))
m_t_sex
```

```
##  contrast estimate   SE   df t.ratio p.value
##  F - M       0.308 0.12 6.77   2.575  0.0378
##
## Results are averaged over the levels of: Sp
## Degrees-of-freedom method: kenward-roger
## Results are given on the log (not the response) scale.
```

```
anova(m_time_ad, m_time_ad2)
```

```
## refitting model(s) with ML (instead of REML)
```

```
## Data: data_ad
## Models:
## m_time_ad2: log(Time) ~ Sex + Sp + Rep + (1 | Subject)
## m_time_ad: log(Time) ~ Sex * Sp + Rep + (1 | Subject)
##            npar    AIC    BIC  logLik -2*log(L)  Chisq Df Pr(>Chisq)
## m_time_ad2    6 689.78 714.62 -338.89    677.78
## m_time_ad     7 690.01 718.98 -338.00    676.01 1.7775  1     0.1825
```
